# Low revision rate despite poor functional outcome after stemmed hemiarthroplasty for acute proximal humeral fractures: 2,750 cases reported to the Danish Shoulder Arthroplasty Registry

**DOI:** 10.1080/17453674.2019.1597491

**Published:** 2019-04-01

**Authors:** Alexander Amundsen, Jeppe V Rasmussen, Bo S Olsen, Stig Brorson

**Affiliations:** a Department of Orthopaedic Surgery, Herlev-Gentofte University Hospital, Herlev;;; b Department of Orthopaedic Surgery, Zealand University Hospital, Køge, Denmark

## Abstract

Background and purpose — The revision rate of stemmed hemiarthroplasty (SHA) for acute proximal humeral fractures is low, but does not necessarily reflect the functional outcome. We report the revision rate of SHA for acute proximal humeral fractures and the proportion of arthroplasties that are not revised despite low functional outcome scores.

Patients and methods — The Danish Shoulder Arthroplasty Registry was used to identify all patients with a proximal humeral fracture that was treated with a SHA between January 1, 2006 and December 31, 2015. Information on demographics, surgical procedures, and revisions was collected by the registry. The Western Ontario Osteoarthritis of the Shoulder (WOOS) index at 1 year was used as functional outcome score. We converted the score to a percentage of a maximum score with 100 being the best.

Results — 2,750 SHAs in 2,719 patients were included. Mean age was 72 years (SD 11); 79% were women. Mean WOOS at 1 year was 55 (SD 26). A total of 101 (4%) arthroplasties were revised, and the 10-year cumulative implant survival rate was 95%. The Cox regression model showed a statistically significant impact on implant survival of age, but not of sex or arthroplasty brand. A WOOS score below 30 and 50 was reported in 11% and 25% of patients, respectively.

Interpretation — We found a high implant survival rate, but also a high proportion of patients with a low functional outcome score 1 year after surgery.

Proximal humeral fractures account for approximately 10% of all fall-related fractures in adults (Court-Brown et al. 2017) and the incidence increases with age, with females over 80 years having an incidence rate of 379 per 100,000 person years (Launonen et al. 2015a). A rise in the incidence of proximal humeral fractures is expected with an increasing elderly population.

Most proximal humeral fractures can be managed non-surgically (Handoll and Brorson 2015, Launonen et al. 2015b). However, in the case of head split fractures or fracture dislocations, an arthroplasty may be considered because of a high risk of avascular necrosis and post-traumatic osteoarthritis. The stemmed hemiarthroplasty (SHA) has traditionally been preferred. It may result in satisfactory long-term pain relief, but results for postoperative shoulder movement have been less predictable (Antuna et al. 2008). The reason for this may be related to impaired rotator cuff function and non-union of the tuberosities (Kralinger et al. 2004, Greiner et al. 2009, Boileau et al. 2013, Giovale et al. 2014, Hashiguchi et al. 2015).

Revision rates after SHA are low, ranging from 1% to 9% (Fevang et al. 2009, Namdari et al. 2013, Brorson et al. 2017). However, revision rates do not necessarily reflect the functional outcome as some arthroplasties are never revised due to patient- or surgery-related factors.

We report the revision rate of SHA for acute proximal humeral fractures and the proportion of arthroplasties that are not revised despite low functional outcome scores.

## Patients and methods

**Figure 1.**
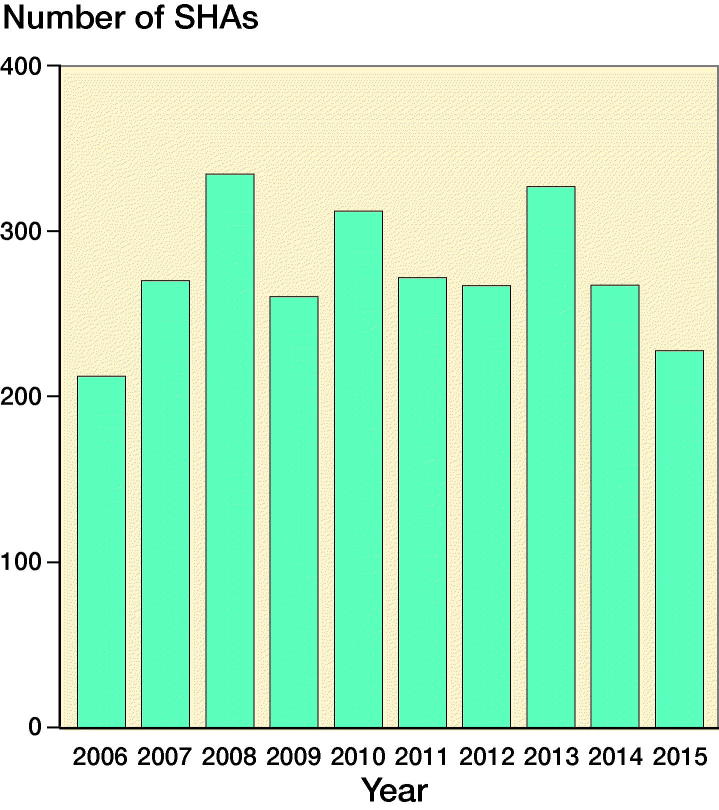
Annual number of SHAs for acute proximal humeral fractures in Denmark from 2006 to 2015.

The Danish Shoulder Arthroplasty Registry (DSR) was used to identify all patients with proximal humeral fractures treated with SHA between January 1, 2006 and December 31, 2015 (Figure 1). Only patients who were surgically managed within 2 weeks from the date of injury were included.

The DSR is a national registry that was established in 2004. All surgeons performing shoulder arthroplasty, at hospitals or private clinics in Denmark, have been obliged to report to the DSR since 2006. Information concerning demographics and the surgical procedure is reported to the registry by the surgeon at the time of the operation (Rasmussen et al. 2012). The 1-year functional outcome is assessed with the Western Ontario Osteoarthritis of the Shoulder (WOOS) index. The WOOS is managed by the DSR, which sends patients a questionnaire 10–14 months after surgery. A single reminder was sent to non-responders. DSR uses the WOOS questionnaire for all patients with shoulder arthroplasties regardless of indication for surgery. Currently, the DSR does not include information on fracture classification or other functional outcome scores in the database.

WOOS is a patient-administrated quality of life questionnaire consisting of 19 questions divided into 4 categories (physical symptoms, sports and work, lifestyle, and emotions). Each question is designed as a visual analogue scale, ranging from 0 to 100 points, with 100 being the worst. In this study the total WOOS scores are converted to percentages, with 100 being the best. A questionnaire was marked as incomplete if 1 or more questions were unanswered. The Danish translated version of WOOS has previously been validated for osteoarthritis patients (Rasmussen et al. 2013), but is yet to be validated for fracture patients.

Patients who died or whose surgery was revised within 1 year of surgery were not sent a WOOS questionnaire. If revision occurred later than 1 year after surgery the WOOS score of the primary arthroplasty procedure was included in the analysis.

A revision was defined as removal or exchange of the hemiarthroplasty component or the addition of a glenoid component. The revision procedure is linked to the primary procedure using a unique civil registration number given at birth. Information regarding the revision procedure including the indication for revision is reported to the registry by the surgeon at the time of the revision procedure. It is possible for the surgeon to report more than 1 indication for revision. In these cases, we used a hierarchy to classify the indication for revisions (Table 1). This was based on a previously reported hierarchy of reasons for revisions made by the Nordic Arthroplasty Register Association (NARA), which has been adapted by the DSR (Rasmussen et al. 2016).

**Table 1.** Hierarchy of reasons for revision where more than one reason was reported

Hierarchy of reasons for revision
I Infection – an infection that requires revision of the arthroplasty
II Periprostethic fracture – fracture that requires revision of the arthroplasty
III Dislocation and instability
IV Loosening – loosening of any arthroplasty component
V Rotator cuff problem
VI Others – glenoid wear, biomechanical problems including overstuffing, and pain with no other complication

The vital status of patients was obtained through the Danish National Register of Persons and linked to the data from the DSR using the civil registration number.

### Statistics

Hazard ratios were calculated using Cox’s proportional hazards regression model with 95% confidence intervals (CI). Age, sex, year of surgery, and arthroplasty brand were included in the model. Log–log plots and Schoenfeld residuals were used to check that the proportional hazards assumption was fulfilled. The 10-year cumulative implant survival rate was illustrated using the Kaplan–Meier method with revision as the endpoint. A log-rank test was used to compare the implant survival rates of different age groups. The 1-way Anova test was used to compare the mean WOOS score of different age groups. A chi-square test was used to compare response rates between men and women.

The presumption of independence is violated when we include bilateral procedures in the survival analyses and, even though this may theoretically have consequences, no practical problems have been shown when analyzing arthroplasty register data (Ranstam et al. 2011). Additionally, the underlying assumption of no competing risks, which forms the basis of the Cox regression model and Kaplan–Meier method, is violated in survival analyses of arthroplasty data as patients are censored if they die.

SPSS version 22.0 (IBM Corp, Armonk, NY, USA) was used to perform the analyses. P-values were 2-tailed and a p-value < 0.05 was set as the level of statistical significance.

### Ethics, funding, and potential conflicts of interest

Permission to handle and store data was obtained from the Danish Data Protection Agency (date 03.07.2014, j.nr. 2007-58-0015). According to the regulations in Denmark, this study did not need permission from the National Committee on Health Research Ethics. There was no conflicts of interest to be declared related to this study.

## Results

2,750 SHAs in 2,719 patients were included; 79% were women and the mean age was 72 years (SD 11) (Table 2). The use of SHA was stable in the period from 2007 to 2012, with peaks in 2008, 2010, and 2013. A decrease in SHA was found from 2013 to 2015 (Figure 1).

**Table 2.** Demographics, proportion of revisions, and mean WOOS score overall and for each age group

	Total	< 55 years	55–74 years	≥ 75 years
Sex, n (%)				
Male	570 (21)	104 (59)	310 (24)	156 (12)
Female	2,180 (79)	73 (41)	1,002 (76)	1,105 (88)
Prosthesis brand, n (%)				
Depuy Global FX	952 (34)	48 (27)	495 (38)	409 (32)
Zimmer Bigliani-Flatow	840 (31)	67 (38)	372 (28)	401 (32)
Tornier Aequalis	268 (10)	22 (12)	141 (11)	105 (8)
Biomet Comprehensive	245 (9)	15 (9)	98 (7)	132 (11)
Others	445 (16)	25 (14)	206 (16)	214 (17)
Revision, n (%)	101 (4)	9 (5)	70 (5)	22 (2)
WOOS				
Complete, n (%)	1,525 (60)	87 (52)	780 (62)	658 (58)
Score, mean (SD)	55 (26)	53 (24)	54 (26)	57 (25)

### WOOS

**Figure 2.**
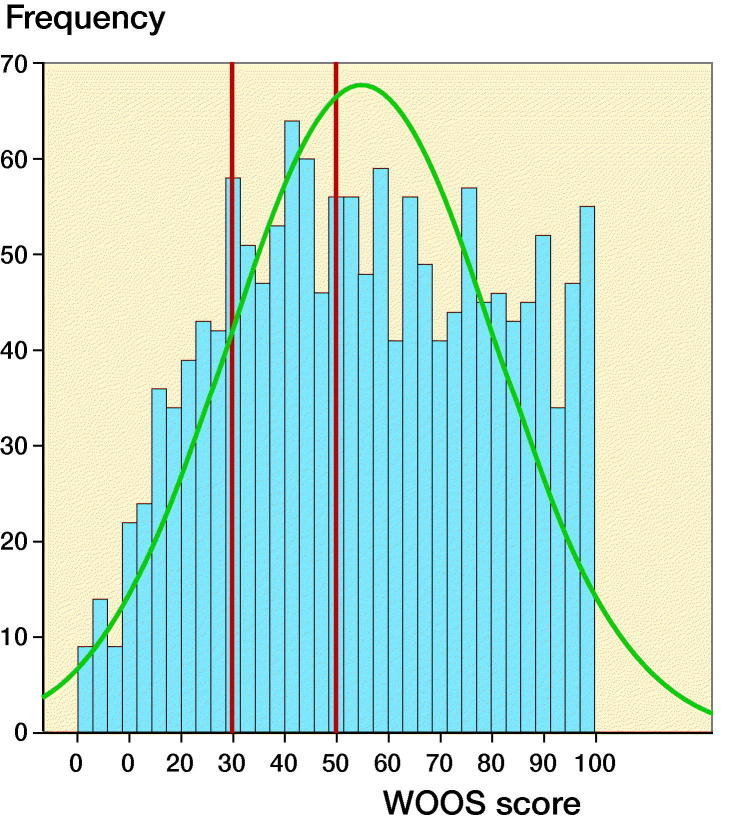
Distribution of WOOS scores. Red lines signify a WOOS score of 30 and 50.

157 (6%) patients died and 37 (1%) patients underwent revision within 1 year of surgery, leaving 2,556 (93%) arthroplasties available for follow-up. WOOS was completed in 1,525 SHAs (60%) with a mean WOOS of 55 (SD 26). Incomplete and missing WOOS questionnaires accounted for 197 (8%) and 834 (33%) respectively. WOOS was completed by 54% male and 61% female patients (p < 0.01). A WOOS score below 30 and 50 was reported in 303 (11%) and 676 (25%) patients, respectively (Figure 2). There were no stastically significant differences in WOOS score between age groups (p = 0.08).

### Revision and prosthesis survival

101 (4%) SHAs were revised. Patients who were younger than 55 and patients between 55 and 74 years had a 2.7 (CI 1.3–5.9) and 2.9 (CI 1.8–4.7) times higher risk of revision compared with patients who were older than 75 years (Table 3). The most common indications for revision were rotator cuff problem (1%) and dislocation (0.8%) (Table 4).

**Table 3.** Risk of revision. Cox regression model^a^

Age	Univariate relative risk (95% CI)	Adjusted relative risk (95% CI)
≥ 75	1.0 (reference)	1.0 (reference)
55–75	2.9 (1.8–4.7)	2.9 (1.8–4.7)
< 55	2.7 (1.3–5.9)	3.3 (1.5–7.5)

a Sex, prosthesis brand (Depuy Global FX, Zimmer Bigliani-Flatow, Tornier Aequalis Fx, Biomet Comprehensive Fx) and year of surgery (2006–2007, 2008–2009, 2010–2011, 2012–2013, 2014–2015) were included in the multiple model and showed no statistical significance.

**Table 4.** Reasons for revision. Values are frequency (percent)

	Total	< 55 years	55–75 years	≥ 75 years
Dislocation	21 (0.8)	2	11	8
Loosening	2 (0.1)	0	2	0
Infection	15 (0.5)	1	11	3
Fracture	1 (0.0)	0	1	0
Rotator cuff failure	30 (1.1)	4	19	7
Others **^a^**	21 (0.8)	2	17	2
Missing	11 (0.4)	0	9	2
Total	101 (3.7)	9	70	22

a Including pain of unknown cause.

**Figure 3.**
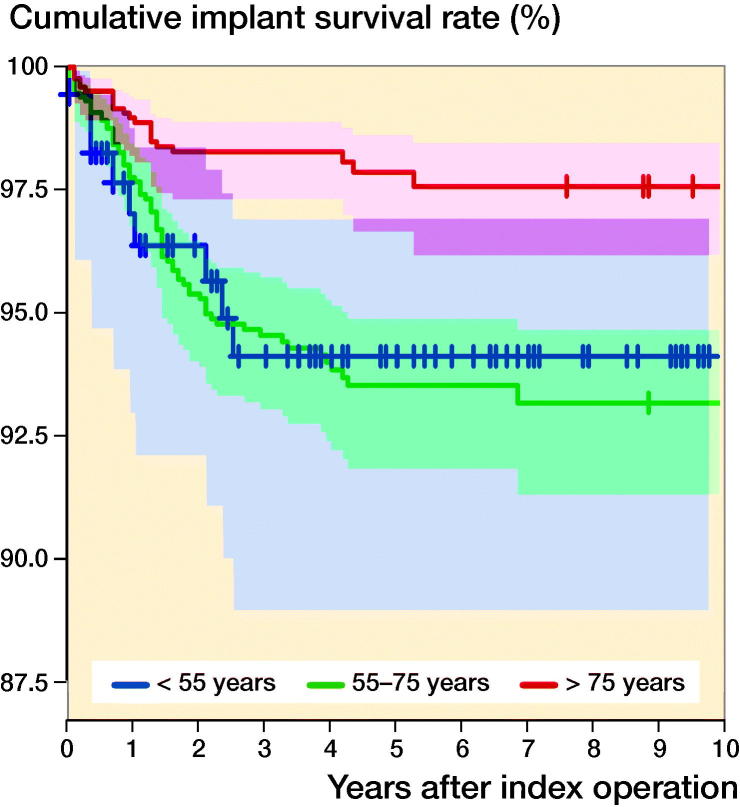
The 10-year cumulative implant survival rate and CI for patients younger than 55 years (blue), patients between 55 and 74 years (green), and patients older than 75 years (red) (p < 0.01).

The 10-year cumulative implant survival rate was 95% (CI 94–96). For patients who were younger than 55 years, patients between 55 and 75 years, and patients who were older than 75 years the survival rates were 94% (CI 89–97), 93% (CI 91–95), and 98% (CI 96–99), respectively (Figure 3).

## Discussion

We found a high implant survival rate, but also a high proportion of patients with a low functional outcome score 1 year after surgery. This adds to the debate concerning the use of SHA and other prostheses in patients with displaced proximal humeral fractures.

Olerud et al. (2011) conducted a randomized controlled trial (RCT), investigating quality of life, function, and pain in 55 patients with displaced 4-part fractures treated non-surgically or surgically with SHA. The patients, with a mean age of 77 years, were followed up 2 years postoperatively. The study found that the health-related quality of life score (EQ-5D 0.81 vs. 0.65), the Disabilities of the Arm, Shoulder and Hand (DASH) score (30 vs. 37), and pain assessment (VAS 15 vs. 25) were in favor of SHA compared with non-surgically treated patients. However, the only statistically significant difference was found in EQ-5D and the score differences in DASH and VAS are too small to be considered clinically significant. Range of motion and Constant score were similar between the 2 groups.

In a RCT comparing non-surgical and SHA-treated Neer 4-part fractures in 50 patients older than 65 years, Boons et al. (2012) found that there was no clear benefit for SHA compared with non-surgical treatment. Constant score and Simple Shoulder Test were similar 3 or 12 months after surgery. Both groups had improved strength 12 months after surgery and the non-surgically treated group had better abduction strength at the 3- and 12-month follow-up compared with SHA-treated patients. However, the non-surgically treated patients experienced more pain than SHA patients at the 3-month follow-up, but this difference was not detectable at 12 months postoperatively. Furthermore a Cochrane review found that patients surgically managed for displaced proximal humeral fractures involving the humeral neck did not have a better outcome compared with non-surgically managed patients 1 and 2 years after surgery (Handoll and Brorson 2015). The evidence of this review did, however, not cover fracture dislocations or head split fractures, as there are no RCTs concerning these indications. In addition, the review found that surgically managed patients were likely to have greater need of subsequent surgery.

The proportion of revisions in our study corresponds with the 3% reported by both the NARA group (Brorson et al. 2017) and in a study of 422 Norwegian patients undergoing SHA for acute proximal humeral fractures (Fevang et al. 2009). In contrast, 9% revisions were found in a systematic review including 7 studies comprising 263 SHAs for proximal humeral fractures. However only 1 study was prospective and only 2 studies had a final follow-up of more than 30 patients (Namdari et al 2013). In our study the most common reason for revision was failure of the rotator cuff. Degeneration or rupture of the rotator cuff or nonunion of the tuberosities are associated with proximal migration, which can be painful and can restrict movement of the shoulder.

The implant survival rate in our study reflects previously reported rates. The NARA group found 1-, 5-, and 10-year implant survival rates of 0.99, 0.96, and 0.95 respectively for SHA used for proximal humeral fractures (Brorson et al. 2017). Another study from the United States (Farng et al. 2011) reported a 10-year cumulative survival rate of 94% for 5,044 patients with proximal humeral fractures. However, 6% of patients were treated with total shoulder arthroplasty. An improvement of implant survival to a final 5-year cumulative survival rate of 95% was found in a study of 751 acute fractures, of which 86% were treated with SHA (Fevang et al. 2015).

The low revision rate is in contrast to the high number of patients reporting a low WOOS score 1 year after surgery. The reason for this is unknown, but surgeons might hesitate to revise because of age, comorbidity, or low functional demands or because the revision procedure can be challenging. Thus, for patients with proximal humeral fractures the revision rate alone does not necessarily reflect the effect of the shoulder arthroplasty. The reporting of satisfaction after SHA for proximal humeral fractures varies in the literature and many studies do not account for the assessment used. The patient satisfaction self-assessment scale was used in a study of 51 patients, with a mean age of 71 years and of whom 39 were female, who were treated with SHA for 3- or 4-part proximal humeral fractures (Valenti et al. 2017). This study found that 13 of 51 patients reported poor satisfaction after 18 months. The age and distribution of female patients in this study is similar to our study. In a study by Boileau et al. (2002), 66 patients were asked if they were very satisfied, satisfied, disappointed, or unhappy with the functional outcome 27 months after SHA for 3- and 4-part proximal humeral fractures. 29 patients were either disappointed or unhappy.

We found differences in WOOS score between age groups, but none were statistically significant. A study on the social implications of SHA for proximal humeral fractures in patients older than 70 years found that 85% of patients lived in their own environment and managed daily life despite poor shoulder function (Dietrich et al. 2007). Furthermore, the difference in functional outcome between younger and elderly patients might be due to differences in general health condition and in the mechanisms of trauma. Most proximal humeral fractures occur in elderly patients with osteoporosis suffering low-energy trauma, while the force causing the fractures among younger patients is higher. Younger patients might still be working or performing activities that require good shoulder function. Thus, the outcome may not meet their expectations.

The strength of this study is the high number of patients and the WOOS questionnaire, providing valuable information on the 1-year functional outcome. The use of data on a national level is associated with high external validity. This study has limitations. The completeness of the WOOS questionnaire was 60%, which leaves a large group of non-responders. A study on the reliability of patient-reported outcome in DSR showed that the non-responders do not appear to bias the overall result (Polk et al. 2013). However, that study was based on all indications for shoulder arthroplasty and the study did not manage to contact 18% of the patients. The DSR does not have information on general health condition, radiographs, or classification of the fractures in the database. We have information only on the patient-reported outcome at 1 year, but the functional outcome might improve after 1 year. The study does not provide information on functional outcome after revision arthroplasties. This is important, as some revisions lead to a good functional outcome and cannot be considered as persisting failures. Lastly, WOOS was invented for patients with osteoarthritis and has not been validated for patients treated with shoulder arthroplasty for proximal humeral fractures.

In summary, we found a high implant survival rate, but also a high proportion of patients with a low functional outcome score 1 year after surgery. The reason for this is unknown, but surgeons might hesitate to revise because of age, comorbidity, or low functional demands or because the revision procedure can be challenging. Young age was associated with an increased risk of revision compared with older patients, but the functional outcome at 1 year was similar.

AA obtained permissions, analyzed data, and prepared the manuscript of this study. AA and JVR did the statistical analyses. Reading and proofing of the manuscript was done by JVR, BSO, and SB.

The authors would like to thank all surgeons in Denmark for reporting to DSR and thank the DSR for providing us with data.

*Acta* thanks Antti Loosi and Sari Ponzer for help with peer review of this study.

## References

[CIT0001] Antuna S A, Sperling J W, Cofield R H Shoulder hemiarthroplasty for acute fractures of the proximal humerus: a minimum five-year follow-up. J Shoulder Elb Surg 2008; 17(2): 202–9. 10.1016/j.jse.2007.06.025 18248746

[CIT0002] Boileau P, Krishnan S G, Tinsi L, Walch G, Coste J S, Mole D Tuberosity malposition and migration: reasons for poor outcomes after hemiarthroplasty for displaced fractures of the proximal humerus. J Shoulder Elb Surg 2002; 11(5): 401–12. 10.1067/mse.2002.124527 12378157

[CIT0003] Boileau P, Winter M, Cikes A, Han Y, Carles M, Walch G, Schwartz D G Can surgeons predict what makes a good hemiarthroplasty for fracture? J Shoulder Elb Surg 2013; 22(11): 1495–506. 10.1016/j.jse.2013.04.018 23834993

[CIT0004] Boons H W, Goosen J H, van Grinsven S, van Susante J L, van Loon C J Hemiarthroplasty for humeral four-part fractures for patients 65 years and older: a randomized controlled trial. Clin Orthop Relat R 2012; 470(12): 3483–91. 10.1007/s11999-012-2531-0 PMC3492647 22895694

[CIT0005] Brorson S, Salomonsson B, Jensen S L, Fenstad A M, Demir Y, Rasmussen J V Revision after shoulder replacement for acute fracture of the proximal humerus. Acta Orthop 2017; 88(4): 446–50. 28350203 10.1080/17453674.2017.1307032 PMC5499339

[CIT0006] Court-Brown C M, Clement N D, Duckworth A D, Biant L C, McQueen M M The changing epidemiology of fall-related fractures in adults. Injury 2017; 48(4): 819–24. 28283181 10.1016/j.injury.2017.02.021

[CIT0007] Dietrich M, Meier C, Zeller D, Grueninger P, Berbig R, Platz A Primary hemiarthroplasty for proximal humeral fractures in the elderly: long-term functional outcome and social implications. Eur J Trauma Emerg Surg 2007; 33(5): 512–19. 26814936 10.1007/s00068-007-6134-5

[CIT0008] Farng E, Zingmond D, Krenek L, Soohoo N F Factors predicting complication rates after primary shoulder arthroplasty. J Shoulder Elb Surg 2011; 20(4): 557–63. 10.1016/j.jse.2010.11.005 21324715

[CIT0009] Fevang B T, Lie S A, Havelin L I, Skredderstuen A, Furnes O Risk factors for revision after shoulder arthroplasty: 1,825 shoulder arthroplasties from the Norwegian Arthroplasty Register. Acta Orthop 2009; 80(1): 83–91. 19297791 10.1080/17453670902805098 PMC2823234

[CIT0010] Fevang B T, Nystad T W, Skredderstuen A, Furnes O N, Havelin L I Improved survival for anatomic total shoulder prostheses. Acta Orthop 2015; 86(1): 63–70. 25386737 10.3109/17453674.2014.984113 PMC4366677

[CIT0011] Giovale M, Mangano T, Roda E, Repetto I, Cerruti P, Kuqi E, Franchin F Shoulder hemiarthroplasty for complex humeral fractures: a 5 to 10-year follow-up retrospective study. Musculoskelet Surg 2014; 98(Suppl 1): 27–33. 10.1007/s12306-014-0319-y 24659196

[CIT0012] Greiner S H, Diederichs G, Kroning I, Scheibel M, Perka C Tuberosity position correlates with fatty infiltration of the rotator cuff after hemiarthroplasty for proximal humeral fractures. J Shoulder Elb Surg 2009; 18(3): 431–6. 10.1016/j.jse.2008.10.007 19157911

[CIT0013] Handoll H H, Brorson S Interventions for treating proximal humeral fractures in adults. Cochrane DB Syst Rev 2015(11): Cd000434. 10.1002/14651858.CD000434.pub4 26560014

[CIT0014] Hashiguchi H, Iwashita S, Ohkubo A, Takai S The outcome of hemiarthroplasty for proximal humeral fractures is dependent on the status of the rotator cuff. Int Orthop 2015; 39(6): 1115–19. 25864089 10.1007/s00264-015-2758-y

[CIT0015] Kralinger F, Schwaiger R, Wambacher M, Farrell E, Menth-Chiari W, Lajtai G, Hubner C, Resch H Outcome after primary hemiarthroplasty for fracture of the head of the humerus: a retrospective multicentre study of 167 patients. J Bone Joint Surg Br 2004; 86(2): 217–9. 15046436 10.1302/0301-620x.86b2.14553

[CIT0016] Launonen A P, Lepola V, Saranko A, Flinkkila T, Laitinen M, Mattila V M Epidemiology of proximal humerus fractures. Arch Osteoporos2015a; 10: 209. 25675881 10.1007/s11657-015-0209-4

[CIT0017] Launonen A P, Lepola V, Flinkkila T, Laitinen M, Paavola M, Malmivaara A Treatment of proximal humerus fractures in the elderly: a systemic review of 409 patients. Acta Orthop2015b; 86(3): 280–5. 25574643 10.3109/17453674.2014.999299 PMC4443467

[CIT0018] Namdari S, Horneff J G, Baldwin K Comparison of hemiarthroplasty and reverse arthroplasty for treatment of proximal humeral fractures: a systematic review. J Bone Joint Surg Am 2013; 95(18): 1701–8. 24048558 10.2106/JBJS.L.01115

[CIT0019] Olerud P, Ahrengart L, Ponzer S, Saving J, Tidermark J Hemiarthroplasty versus nonoperative treatment of displaced 4-part proximal humeral fractures in elderly patients: a randomized controlled trial. J Shoulder Elb Surg 2011; 20(7): 1025–33. 10.1016/j.jse.2011.04.016 21783385

[CIT0020] Polk A, Rasmussen J V, Brorson S, Olsen B S Reliability of patient-reported functional outcome in a joint replacement registry: a comparison of primary responders and non-responders in the Danish Shoulder Arthroplasty Registry. Acta Orthop 2013; 84(1): 12–17. 23343374 10.3109/17453674.2013.765622 PMC3584596

[CIT0021] Ranstam J, Karrholm J, Pulkkinen P, Makela K, Espehaug B, Pedersen A B, Mehnert F, Furnes O Statistical analysis of arthroplasty data, I: Introduction and background. Acta Orthop 2011; 82(3): 253–7. 21619499 10.3109/17453674.2011.588862 PMC3235301

[CIT0022] Rasmussen J V, Jakobsen J, Brorson S, Olsen B S The Danish Shoulder Arthroplasty Registry: clinical outcome and short-term survival of 2,137 primary shoulder replacements. Acta Orthop 2012; 83(2): 171–3. 22329671 10.3109/17453674.2012.665327 PMC3339532

[CIT0023] Rasmussen J V, Jakobsen J, Olsen B S, Brorson S Translation and validation of the Western Ontario Osteoarthritis of the Shoulder (WOOS) index: the Danish version. Patien Relat Outcome Meas 2013; 4: 49–54. 10.2147/PROM.S50976 PMC3796868 24133377

[CIT0024] Rasmussen J V, Brorson S, Hallan G, Dale H, Aarimaa V, Mokka J, Jensen S L, Fenstad A M, Salomonsson B Is it feasible to merge data from national shoulder registries? A new collaboration within the Nordic Arthroplasty Register Association. J Shoulder Elb Surg 2016; 25(12): e369–e77. 10.1016/j.jse.2016.02.034 27107732

[CIT0025] Valenti P, Aliani D, Maroun C, Werthel J D, Elkolti K Shoulder hemiarthroplasty for proximal humeral fractures: analysis of clinical and radiographic outcomes at midterm follow-up: a series of 51 patients. Eur J Orthop Surg Tr 2017; 27(3): 309–15. 10.1007/s00590-017-1927-7 28349211

