# Erratum

**DOI:** 10.1080/17453674.2019.1676517

**Published:** 2019-10-15

Low revision rate despite poor functional outcome after stemmed hemiarthroplasty for acute proximal humeral fractures: 2,750 cases reported to the Danish Shoulder Arthroplasty Registry

Alexander AMUNDSEN et al.

Correspondence: alexander.amundsen.03@regionh.dk

Results

….

WOOS

**Erronous text:**

…. A WOOS score below 30 and 50 was reported in 303 (**11%**) and 676 (**25%**) patients, respectively (Figure 2).

These percentages have been calculated using the total number of cases in the study (2,750) and not by using the number of patients whom answered WOOS (1,525). The correction does not affect other sections of the article or change the conclusion.

**The corrected text should be:**

…. A WOOS score below 30 and 50 was reported in 303 (**20%**) and 676 (**44%**) patients, respectively (Figure 2).

